# P33 Day 3 reviews led by pharmacy technicians—encouraging antimicrobial stewardship

**DOI:** 10.1093/jacamr/dlac004.032

**Published:** 2022-02-16

**Authors:** Shazia Nazir

**Affiliations:** Leeds Teaching Hospitals NHS Trust, Leeds, UK

## Abstract

**Background:**

A documented 72 h antimicrobial review is a national target that we are expected to achieve at the Leeds Teaching Hospital. Current practice within LTHT provides a pharmacist-led antimicrobial stewardship ward round where IV antimicrobials are reviewed at Day 3 (72 h). Patients prescribed IV antimicrobials are reviewed within 72 h and a decision is made to continue, stop, switch to oral or refer to the outpatient parenteral antimicrobial therapy (OPAT) service. The Healthcare Associated Infection (HCAI) Annual Programme for this year highlights the importance of expanding antimicrobial stewardship within the pharmacy team. This presented an opportunity to develop a new antimicrobial stewardship strategy by leading a virtual pharmacy technician-led antimicrobial stewardship ward round. This allowed for a greater number of patients that were prescribed IV antimicrobials to reviewed and the potential for less patients to remain on broad-spectrum IV antimicrobials for extended durations.

**Problem:**

Clinicians and pharmacists struggle to undertake this review due to the demands of the daily ward rounds and other reviews that take priority that are needed to ensure patients safety. The electronic prescribing system used within the trust prompts users to undertake Day 3 reviews; however, this is not utilized. This often leads to a greater number of patients remaining on broad-spectrum IV antimicrobials, alongside a lack of justification for remaining on IV antimicrobials despite robust, easily accessible guidelines, education, training and electronic systems.

**Assessment of problem and analysis of causes:**

Firstly, it is a large teaching hospital that has a variation of different specialities and departments, a lot of patients and fewer people to do the reviews and large turnover of staff and patients. Antimicrobial stewardship is complex, everybody has a role to play in making sure that we use antibiotics appropriately and effectively as IV antibiotics can delay discharge, they are costly, the patients are at risk of line infection, they require IV access and they require more nursing time, so it is necessary to embed antimicrobial stewardship good practice principles throughout the trust and promote that everyone needs to play an active role in this. On identifying the problem, I gained feedback from the pharmacy technicians as to why this review could not be done by them; they reported a lack of knowledge of how to review eligibility of patients for oral switch and with the appropriate training this would be something that they would be happy to do within their daily tasks.

**Methods:**

One pharmacy technician (specializing with infectious disease/pharmacy infection team) was appropriately trained to complete virtual reviews, utilizing an electronic LTHT IV antimicrobial reviewing tool which is available to upload to the patient's electronic records. Patients were identified using a daily antimicrobial prescribing list and filtered for those that had been prescribed IV antimicrobials for 3 days. This allowed for a variety of specialities to be included within the audit. Inclusion criteria: patients aged >16 years, inpatients prescribed IV antibiotics for 72 h. Exclusion criteria: patient aged <16 years, antimicrobial prescribed for the treatment of cystic fibrosis, patients prescribed directed antimicrobial therapy based on an infection specialist recommendation (not empirical treatment), those on oral antibiotics, prophylactic antimicrobials. Data were collected throughout the improvement projected as a baseline audit to understand the impact the intervention made, and results were collated and recorded into an Excel spreadsheet and processed:

**Results:**

A total of 38 patients were included in the audit as having had IV antimicrobial reviews completed. Of these, 28 patients fitted the criteria for a switch from IV to oral antimicrobials and therefore recommendations reflecting this were documented. Advice to de-escalate to oral antimicrobials was followed for 20 patients resulting in a hospital discharge after 24 h. The other 10 patients were advised to continue IV treatment until more clinically stable (advice was followed for 9 patients). Overall recommendations were followed in 76% of cases.

**Conclusions:**

Overall, pharmacy technician-led antimicrobial virtual reviews have shown a positive impact on antimicrobial stewardship, by reducing the number of inappropriate IV antimicrobial continued beyond 3 days and ensuring that a greater number of clinical reviews have been documented for the empirical prescribing of IV antimicrobials. This project highlights the potential for a greater number of pharmacy technicians to get involved with IV antimicrobial reviews (once appropriately trained) to complete a small number of reviews weekly, which in turn could provide a reduction of inpatient bed days, decrease harm to patients through the prescribing of antimicrobials, decrease medication costs and improve patient care by facilitating discharge and safeguarding antimicrobials. Additionally, this could help to upskill staff, allowing for greater job satisfaction alongside improved antimicrobial stewardship within the trust.

